# Development of Clarstatin, a Novel Drug Lead for the Therapy of Autoimmune Uveitis

**DOI:** 10.3390/pharmaceutics16060723

**Published:** 2024-05-27

**Authors:** Shira Merzbach, Amnon Hoffman, Philip Lazarovici, Chaim Gilon, Radgonde Amer

**Affiliations:** 1 Institute for Drug Research, School of Pharmacy, Faculty of Medicine, The Hebrew University of Jerusalem, Jerusalem 9112001, Israel; shira.zuriya@mail.huji.ac.il (S.M.); philipl@ekmd.huji.ac.il (P.L.); 2 Institute of Chemistry, The Hebrew University of Jerusalem, Jerusalem 9190401, Israel; chaimgilon@gmail.com; 3 Department of Ophthalmology, Hadassah Medical Center, Faculty of Medicine, Hebrew University of Jerusalem, Jerusalem 9112001, Israel; radgonde@gmail.com

**Keywords:** backbone cyclization, thiourea-bridged, peptomer, calreticulin, experimental autoimmune uveoretinitis, inflammatory autoimmune diseases, peptidomimetic, uveitis

## Abstract

We describe the design, synthesis, and activity of a potent thiourea-bridged backbone cyclic peptidomimetic known as Clarstatin, comprising a 5-amino acid sequence (Q/D)^1^-(R/K)^2^-X^3^-X^4^-A^5^-(Gln/Asp)^1^-(Arg/Lys)^2^-AA^3^-AA^4^-Ala^5^-based on a motif called “shared epitope (SE)”, specifically present in specific alleles of the *HLA-DRB1* gene. This SE binds to a particular site within the proline reach domain (P-domain) of the cell surface-calreticulin (CS-CRT). CS-CRT is a multifunctional endoplasmic reticulum (ER) calcium-binding protein that is located on the cell surface of T cells and triggers innate immune signaling, leading to the development of inflammatory autoimmune diseases. The development of Clarstatin was based on the parent peptide W-G-D^1^-K^2^-S^3^-G^4^-A^5^- derived from the active region of the SE. Following the design based on the cycloscan method, the synthesis of Clarstatin was performed by the Fmoc solid phase peptide synthesis (SPPS) method, purified by HPLC to 96% homogeneity, and its structure was confirmed by LC-MS. Clarstatin reduced calcium levels in Jurkat lymphocyte cultures, ameliorated uveitis in vivo in the experimental autoimmune uveitis (EAU) mice model, and was safe upon acute toxicity evaluation. These findings identify Clarstatin as a promising lead compound for future drug development as a novel class of therapeutic agents in the therapy of uveitis.

## 1. Introduction

Uveitis is an ocular sight-threatening inflammation that affects the uveal tract (iris, choroid, and ciliary body) and may also affect the adjacent structures (including the sclera, cornea, vitreous humor, retina, and optic nerve head). Some uveitic entities may be chronic or recurrent, thus causing transient or permanent visual impairment and ocular complications. Uveitis can occur either as a co-manifestation of various autoimmune disorders and infections or it can arise as purely idiopathic ocular inflammation [1]. The management of uveitis remains a challenge for clinicians, in particular, because of the paucity of specific eye-targeted anti-inflammatory drugs. Despite the multitude of adverse effects, corticosteroids remain the first-line and the mainstay of therapy for patients with active uveitis [2]. Therapeutics targeting common inflammatory pathways are also used. These treatments include steroid-sparing immunomodulatory agents. Although often effective, these agents can be associated with potentially severe adverse events [3]. Therefore, there is an unmet clinical need to develop new, safe, and effective drugs for uveitis.

Population stratification investigations have linked autoimmune diseases with polymorphism and mutations of alleles of the major histocompatibility complex (MHC) system called the human leukocyte antigen (HLA). HLA isotypes belonging to MHC class II are HLA-DP, HLA-DM, HLA-DOA, HLA-DOB, HLA-DQ, and HLA-DR [4]. Despite the highly polymorphic nature of the human leukocyte antigens (HLAs) class II genes, the majority of autoimmune diseases are linked to a limited set of class *II-DR* or -*DQ* gene alleles polymorphism and/or mutations such as the *DRB1*, *DQB1*, and *DPB1* [5]. Genetic studies have linked uveitis to certain MHC class II alleles such as *HLA-DR4* [6]. HLA genes show a strong association with both Vogt–Koyanagi–Harada (VKH) (*HLA-DR4*, *DRB1/DQA1*) and Behcet’s disease (BD) (*HLA-B51*), which are two multi-systemic diseases that may present with non-infectious uveitis [7]. These alleles bear a similar amino acid sequence within the DRB1 molecule, generating the “shared-epitope (SE)” hypothesis [8]. This hypothesis claims that possession of the common, five-residue sequence motif in the DR1β-chain may confer an increased risk for autoimmune diseases, including uveitis [9]. For this reason, the (Q/D)^1^-(R/K)^2^-X^3^-X^4^-A^5^ five-amino acid SE consensus motif was proposed as essential and sufficient to confer susceptibility to an autoimmune disease, as exemplified in the therapy of rheumatoid arthritis [10]. The native conformation of this sequence is an α-helix and therefore, using cyclic peptides to stabilize this conformation, may produce a potent SE peptidomimetic with drug-like properties. Indeed, several studies developed potent peptidomimetics bearing the SE pharmacophores DKCLA, QKCLA, and DERAA for the therapy of rheumatoid arthritis [10,11,12], but no studies were directed for the development of a cyclic peptide drug for the therapy of uveitis.

Calreticulin (CRT) is a highly conserved calcium-binding protein in hematopoietic cells. In various autoimmune diseases, CRT migrates and binds to the cell surface (CS-CRT) [13] being expressed on human T lymphocytes where it is physically associated with a pool of different molecules such as the MHC [14,15]. The HLA-DR ‘shared epitope’ sequence represents a signal transduction target that binds to CS-CRT [16], activating the innate immune signaling and thus contributing to T lymphocyte activation and inflammatory autoimmunity [17]. The presentation of peptide antigens to T cells by MHC class II proteins is a central process in cellular and humoral immune responses. Among the most highly upregulated proteins in uveitis are calreticulin (CRT) and the HLAs [18]. Therefore, inhibition of the interaction between the disease-associated HLAs (such as DR1 and DR4) and CS-CRT may be useful for the treatment of various autoimmune diseases, including uveitis. In addition, it was suggested that there are reciprocal functional interactions between CS-CRT, integrins, and calcium channels on the cell surface of T cells [19,20], and therefore, inhibition of the signal transduction of CS-CRT may block a wide array of cellular responses critical in immune response [21], thereby providing therapy to a wide range of inflammatory autoimmune diseases. However, peptidomimetic inhibitors of the HLA SE motif-CS-CRT interaction that are effective in the therapy of uveitis were not yet reported, and therefore, these were developed in the present study.

**Figure 1:**
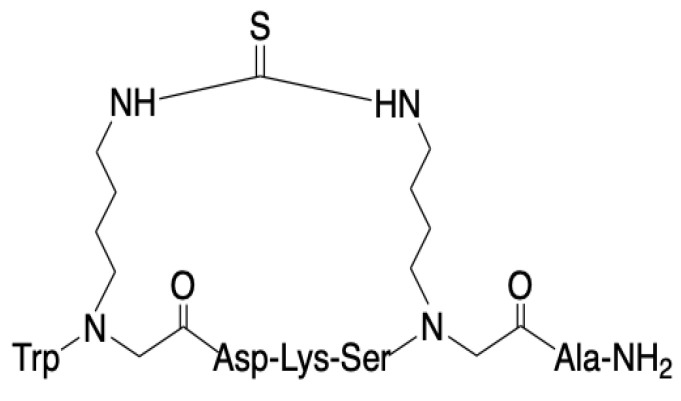
Structure of Clarstatin.

Here we describe the use of a urea-bridged backbone cyclic SE peptidomimetic to design and develop a thiourea-bridged backbone cyclic peptidomimetic analog called Clarstatin (Figure 1). The thiourea bond in thiourea-bridged cyclic peptides was previously used to generate a plethora of guanidine and substituted guanidine’s containing bridges to test the influence of the bridge chemistry on the activity and selectivity of cyclic peptides [22]. A cyclized enkephalin with a thiourea bridge and methyl guanidine bridge showed good biological activity [23]. We hypothesized, therefore, that thiourea-bridged Clarstatin will also be biologically active and represent a pragmatic novel therapeutic strategy for uveitis. We found that Clarstatin reduced calcium levels in Jurkat lymphocyte cultures, ameliorated uveitis-induced eye pathology in an in vivo experimental autoimmune uveitis (EAU) mice model, and was well tolerated upon acute systemic delivery, representing a novel lead compound for the therapy of uveitis.

## 2. Experimental Method

### 2.1. Fluorescent Ca^2+^ Imaging

Jurkat cells were attached to polylysine-coated glass coverslips for Ca^2+^ imaging conducted in Ringer’s solution composed of 126 mM NaCl, 5.4 mM KCl, 0.8 mM MgCl_2_, 20 mM HEPES, 1.8 mM CaCl_2_, and 15 mM glucose. The pH of the solution was adjusted to 7.4 using NaOH. Prior to imaging, the cells were loaded with Fura 2 AM from Teflabs, Jackson Springs, NC, USA. Intracellular Ca^2+^ responses were observed in the presence of 2 mM EGTA. Illumination of cells was accomplished using a 175 W xenon arc lamp, and the excitation wavelengths of 340/380 nm were selected via a Lambda DG-4 monochromatic wavelength changer from Sutter Instrument, Novato, CA, USA. The intracellular Ca^2+^ concentration was quantified using digital video microfluorometry employing a front-illuminated interline CCD camera (Exi Blue; QImaging, Surrey, BC, Canada) alongside MetaFluor Fluorescence Ratio Imaging Software (Meta Imaging Series 6.1) from Molecular Devices, Sunnyvale, CA, USA. Dual images (340 and 380 nm excitation, 510 nm emission) were captured, and pseudocolor ratio-metric images were recorded every 2 s throughout the experiment, all performed at room temperature [24].

### 2.2. Experimental Autoimmune Uveitis Model

#### 2.2.1. Experimental Autoimmune Uveitis Induction

To evaluate the therapeutic effect of Clarstatin, we performed experiments on C57BL/6J mice in an experimental autoimmune uveitis (EAU) mice model. Female, 6- to 8-week-old C57BL/6J mice were maintained in the specific pathogen-free unit of our Faculty of Medicine, and all experiments were approved by the Hebrew University–Hadassah Institutional Animal Care and Use Committee. The mice were immunized subcutaneously (SC) with 500 µg interphotoreceptor retinoid-binding protein (IRBP_1–20_, GPTHLFQPSLVLDMAKVLLD) (Adar Biotech, Rehovot, Israel) emulsified with an equal volume of complete Freund’s adjuvant (CFA) (Sigma, St. Louis, MO, USA) in a total volume of 200 µL. The mixture contained 2.5 mg/mL *Mycobacterium tuberculosis* H37RA (BD, Bethesda, MD, USA). An additional intraperitoneal injection of 1 µg of purified Bordetella pertussis toxin (PTX) (List biological laboratories, Campbell, CA, USA) was also applied to each animal. The control mice were immunized with the same volume of PBS instead of IRBP in CFA and PTX. The mice were sacrificed at day 36 after primary immunization.

#### 2.2.2. Treatment with Clarstatin

Each mouse in the treatment group received 3.6, 36, or 360 μg/kg of Clarstatin in a volume of 50 µL once or twice per week by an intraperitoneal (i.p.) injection (*n* = 4, 8, 5, respectively). The first dose was administered concurrently with EAU induction. The mice in the control group received PBS i.p. at the same volume. In each mouse, one eye was collected and embedded in paraffin for histopathological analysis.

#### 2.2.3. Histological Evaluation of Eye Slices

To analyze the histopathology results after immunization for 36 days, eyes were collected immediately after exitus and prefixed for 24 h in Davidson solution. Next, the fixed eyes were dehydrated in alcohol with concentration gradients and embedded in paraffin. Then, tissue sections (3–6 μm) were stained with hematoxylin and eosin. The severity of uveitis was evaluated histologically and graded in a masked fashion. This grading system permitted a semi-quantitative assessment of the severity and extent of both infiltrative and structural/morphologic changes of the uveitis at various points throughout the course of EAU. Histological changes were evaluated and graded on a scale from 0 to 4 according to the previously described criteria [25].

### 2.3. Cell Death Assay

Cell death of Jurkat cells was measured by the release of lactate dehydrogenase (LDH) into the medium, in the absence and presence of different concentrations of Clarstatin after 48 h of treatment, using the LDH reagent. H_2_O_2_-treated cells were used as a positive control. LDH activity was determined spectrophotometrically at 340 nm by following the rate of conversion of oxidized NAD to the reduced form of NAD (NADH). LDH release was expressed as the optical density units and calculated as a percentage of total LDH. Each experiment was performed three times in six replicates (*n* = 18) [26].

### 2.4. Acute Toxicity Evaluation of Clarstatin in Mice

Female ICR mice (*n* = 5) were injected intravenously with 0.2 mL Clarstatin at a dose of 10 mg/kg. Untreated mice (*n* = 5) were the control. The brain, liver, kidneys, spleen, heart, lungs, small and large intestines, stomach, and thymus from the mice were harvested after 48 h and fixed in 4% formaldehyde. Then, the tissues were trimmed, placed in embedding cassettes, and processed routinely for paraffin embedding. Six cassettes were prepared per animal (8 organs). Paraffin sections (4 microns thick) were cut, placed on glass slides, and stained with haematoxylin and eosin (H&E) for histological evaluation. Pictures were taken with an Olympus microscope (BX60, serial NO. 7D04032) (Olympus, Tokyo, Japan) using the microscope’s camera (Olympus DP73, serial No. OH05504) at an objective magnification of ×10 and ×4. The H&E-stained slides were examined and scored by the study’s pathologist, using a semi-quantitative 5-point grading scale, for the severity of the histopathological changes: Grade 0—the tissue appears normal, without any changes at all; Grade 1—minimal pathological findings; Grade 2—mild pathological findings; Grade 3—moderate pathological findings; Grade 4—severe pathological findings. The histopathological evaluation included a comparison between treated and naïve animals.

### 2.5. Statistics

Data are presented as the mean ± standard error of the mean (SEM) and were considered significant when the *p*-value was <0.05. Statistical analysis was performed using one-way ANOVA followed by Dunnett’s multiple comparisons by using GraphPad Prism 5.0 (GraphPad Software, San Diego, CA, USA).

## 3. Results

### 3.1. SPPS of Clarstatin

**Scheme 1:**
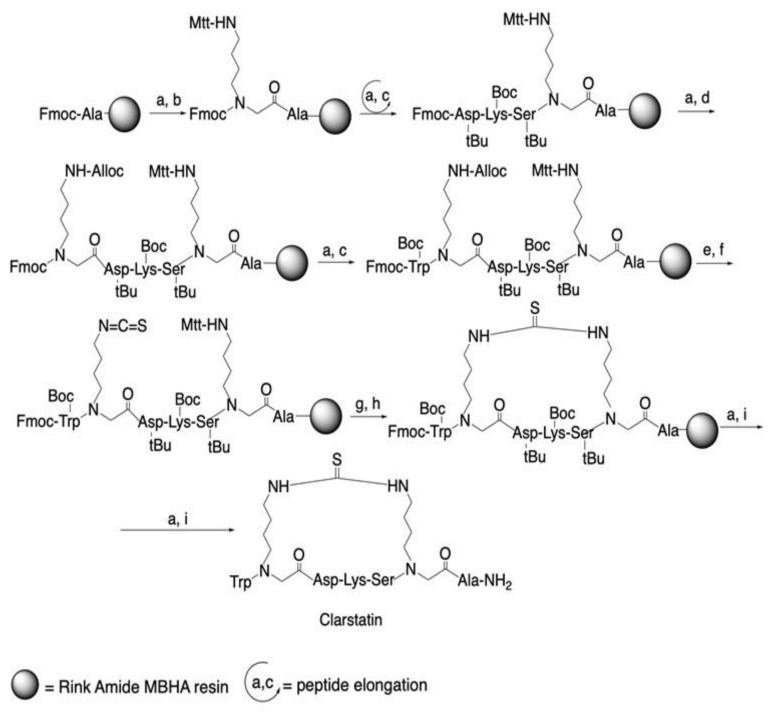
Solid phase synthesis of Clarstatin. Reagents: (a) piperidine/DMF 20%; (b) coupling of Fmoc-[N-(Mtt)δ-aminobutyl]glycine building unit: TBTU/DIEA/DMF; (c) FmocAA + TBTU/DIEA/DMF; (d) coupling of Fmoc-[N-(alloc)δ-aminobutyl]glycine building unit: TBTU/DIEA/DMF; (e) (PPh3)4Pd(0)/N-methylmorpholine/AcOH; (f) isothiocyanate formation: CS_2_/HBTU/DIEA/DMF; (g) DCM/TFE/AcOH (7/2/1); (h) DIEA/DMF; (i) TFA/TIS/H2O (95/2.5/2.5).

The solid phase peptide synthesis of Clarstatin is shown in Scheme 1.

**Figure 2:**
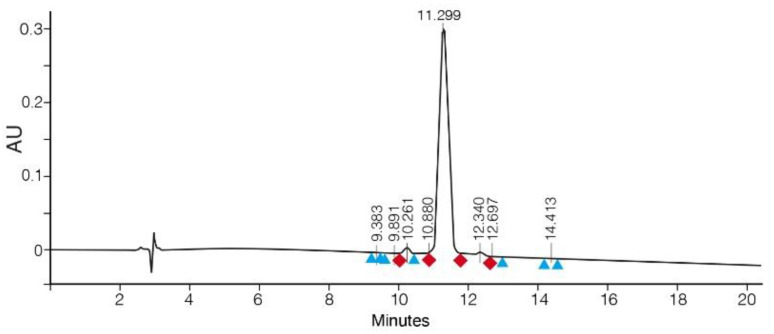
Analytical HPLC of Clarstatin.

**Figure 3:**
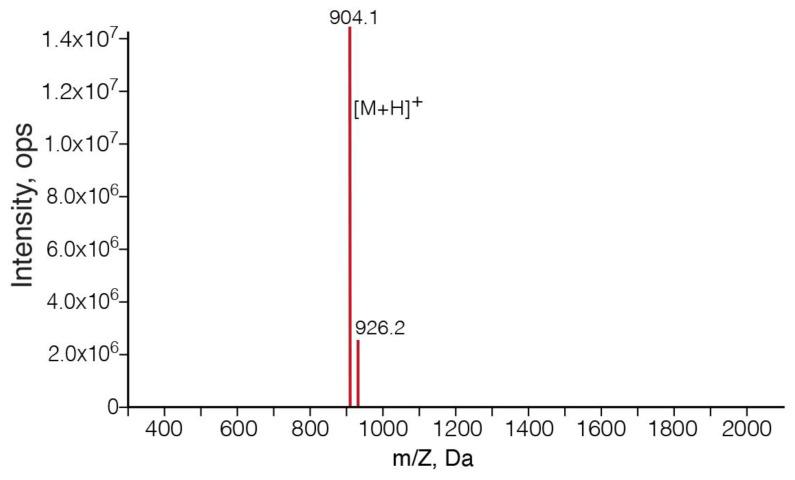
Mass spectrum of Clarstatin.

After synthesis, the crude Clarstatin was purified to 96% homogeneity using preparative HPLC (Figure 2). The molecular structure was then verified by mass spectrometry (Figure 3). In the analytical HPLC, the main peak corresponded to Clarstatin with a molecular weight of 903.1, which matched the calculated value of 903.

### 3.2. Ca^2+^ Signaling Was Reduced in Jurkat Cells upon Clarstatin Treatment

**Figure 4:**
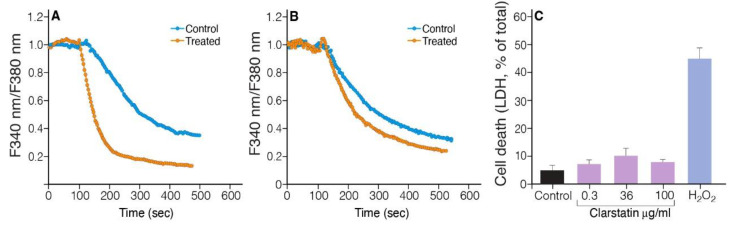
Ca^2+^ signaling was reduced in Jurkat cells upon Clarstatin treatment in the absence of cytotoxic effects. Changes with time of Ca^2+^ signaling in Jurkat cells in response to (**A**) LPS (50 µg/mL) and (**B**) Clarstatin (36 µg/mL); (**C**) necrotic cell death measured by LDH release in Jurkat cells exposed for 48 h to different Clarstatin concentrations; H_2_O_2_ treatment was used as a positive cytotoxic control. Values expressed as mean ± SEM (*n* = 18). *p* ≤ 0.05 for H_2_O_2_ vs. control are percent of LDH released in the medium out of the total culture LDH. No significance was found for Clarstatin concentrations compared to control (ANOVA test).

Multiple inflammatory stimuli converge on Ca^2+^ signaling in immune cells. We sought to characterize the effect of Clarstatin on Ca^2+^ signaling in Jurkat cells in vitro by comparing it to the inflammatory lipopolysaccharide bacterial endotoxin (LPS). Cytosolic intracellular Ca^2+^ content was measured in Jurkat cells loaded with Fura-2AM in the presence of EGTA to prevent extracellular influx by calcium channels. As expected, intracellular Ca^2+^ levels significantly decreased upon LPS treatment (Figure 4A). In the presence of Clarstatin, intracellular Ca^2+^ content was also significantly attenuated (Figure 4B). The overall calcium level, calculated as the area under the curve (AUC), was reduced by 65% in LPS-treated cells and by 15% in Clarstatin-treated cells (orange traces) compared to control-untreated cells (blue traces). Overall, the reduced calcium levels remained significantly decreased for about 500 s. To evaluate a potential cytotoxic effect, Jurkat cells were incubated with 0.3, 36, and 100 µg/mL Clarstatin for 48 h and thereafter the necrotic cell death was measured by the release of LDH. Figure 4C indicates that at in all concentrations, Clarstatin did not significantly increase cell death compared to control-untreated cells, stressing the cellular safety of the Jurkat cells treated with Clarstatin and indicating that the effect of Clarstatin on the intracellular Ca^2+^ content was not due to cytotoxicity.

### 3.3. The Severity of EAU Was Reduced in Mice upon Clarstatin Treatment

To evaluate the therapeutic effect of Clarstatin, we performed experiments on C57BL/6J mice in the EAU mice model. The mice were immunized subcutaneously with the interphotoreceptor retinoid-binding protein (IRBP) to induce uveitis, treated or untreated with Clarstatin, and sacrificed. To analyze the eye histopathology, the eyes were collected, fixed, and eye sections were stained with hematoxylin and eosin. The intensity of eye uveitis was evaluated histologically permitting a semi-quantitative assessment of the severity and extent of the inflammation and pathological changes of the eye. The EAU histopathological score (a scale from 0–4 according to the extent of inflammation and tissue damage) was used for grading the severity of uveitis disease. Score 0—few (1–2) very small, peripheral, focal, chorioretinal lesions and minimal vasculitis; score 1—mild vasculitis, small focal chorioretinal lesions (no more than five), linear chorioretinal lesion (no more than one); score 2—multiple (more than five) chorioretinal lesions and/or inflammatory infiltrates severe vasculitis (large, thick infiltrates), linear chorioretinal lesions (more than five); score 3—pattern of linear chorioretinal lesions, large, confluent chorioretinal lesions, subretinal neovascularization, hemorrhages; score 4—large retinal detachment, retinal atrophy.

**Figure 5:**
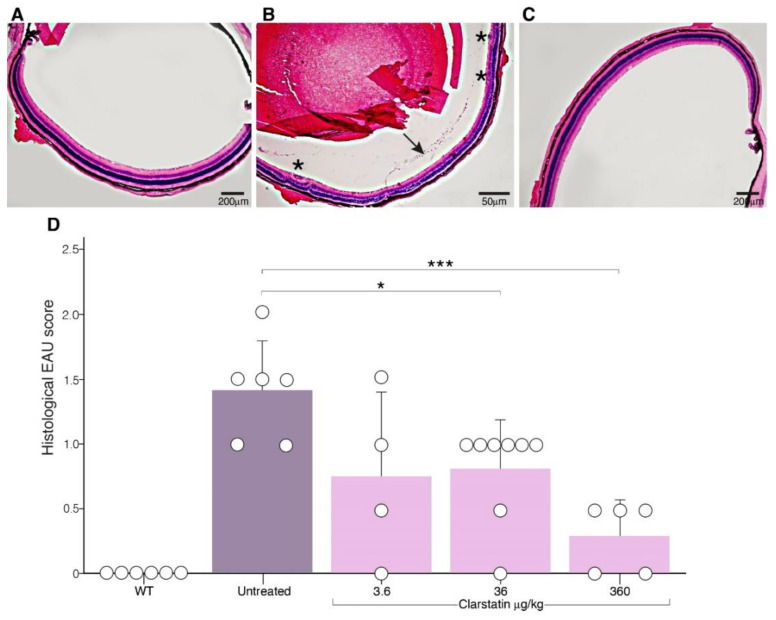
Examination of the therapeutic effect of the cyclic peptide, Clarstatin, in the EAU mice model by histopathology analysis. Representative photographs of the histopathology of retinal sections of C57BL/6J mice 36 days after EAU induction. (**A**) The wild-type mouse, which was not immunized with IRBP, showed normal retinal structure. (**B**) The EAU mouse which was immunized with IRBP showed active uveitis with vitreous cells (arrow) and several foci of retinal infiltrates and vasculitis (star). (**C**) The EAU mouse which was immunized with IRBP and treated with Clarstatin (3.6 μg/kg mice, 50 μL, i.p., twice per week) showed no signs of active uveitis. (**D**) Quantitation by dose response of the therapeutic effect of Clarstatin based on the EAU score from histopathological sections. EAU scores were assigned on a scale from 0 to 4 according to the extent of inflammation and tissue damage. Each point represents one mouse. The ocular inflammatory response was ameliorated in mice treated with Clarstatin compared to untreated mice. * *p* < 0.05 *** *p* < 0.001 (ANOVA test).

The histology sections of the eyes in the treatment group with Clarstatin (3.6 μg/kg, i.p) showed reduced signs of active uveitis (Figure 5C). However, EAU mice that were not treated showed signs of active disease like vitreous cells and several foci of retinal infiltrates and vasculitis (Figure 5B). The wild-type mouse, which was not immunized with IRBP, showed a normal retinal structure (Figure 5A). Quantitation of the therapeutic effect of Clarstatin based on the EAU score from histopathological sections showed that untreated mice developed EAU at an average score of 1.4. Control mice that were not immunized with IRBP (WT) did not develop EAU (score 0). Mice treated with 3.6 µg/kg Clarstatin developed EAU at an average score of 0.7. Mice treated with 36 µg/kg Clarstatin developed EAU at an average score of 0.8. Mice treated with 360 µg/kg Clarstatin developed EAU at an average score of 0.3. In the treatment group which received a dose of 3.6 µg/kg Clarstatin, the EAU score was reduced from 1.4 to 0.7 (*p* = 0.06). In the treatment group which received a dose of 36 µg/kg Clarstatin, the EAU score was reduced from 1.4 to 0.8 (*p* < 0.05). In the treatment group which received a dose of 360 µg/kg Clarstatin, the EAU score was reduced from 1.4 to 0.3 (*p* < 0.01) (Figure 5D).

### 3.4. Acute Tolerability of Clarstatin in Mice without Short-Term Adverse Pathological Effects on Major Organs

**Figure 6:**
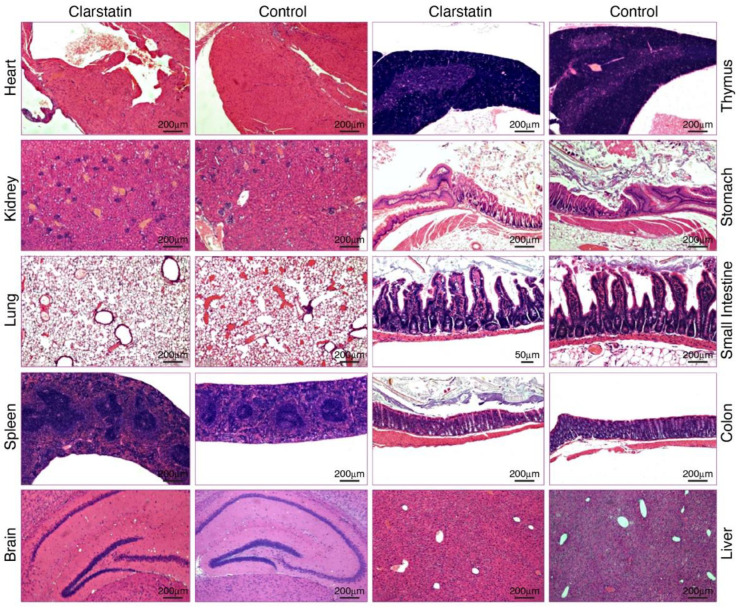
Representative histological images of organ slices stained with hematoxylin and eosin in an acute toxicity test of female ICR mice after administration of Clarstatin (10 mg/kg) by i.v. injection for 48 h.

We conducted an acute, single-dose toxicity study to determine the short-term adverse pathological effects of Clarstatin on major mice organs when administered in mice in a single high dose. One group of five mice received a dose of 0.25 mg/mice (10 mg/kg) Clarstatin by intravenous (i.v.) injection, in a volume of 0.2 mL/mouse. The second group of five naïve mice did not receive Clarstatin and were considered as the control. Before Clarstatin administration, the body weight of each animal was determined, and the dose was calculated according to the body weight. The mice were observed for any toxic effect for the first 4 h after Clarstatin injection and for the entire period of the two days. They did not display any changes in behavior, breathing, skin effects, body weight, urinations, food and water intake, temperature, and changes in eye and skin colors. There were no signs of constipation, sedation, convulsion, tremor, lethargy, drowsiness, coma, or death. After 48 h of exposure and follow up, the animals were sacrificed, and organs were harvested for pathology analyses. By comparing the organs from Clarstatin-treated mice to the control, no pathological changes were observed, as evidenced from Figure 6. The tested organs, including the brain, kidneys, spleen, heart, lungs, small intestine and large intestine, stomach, and thymus looked normal, without any microscopically pathological changes. The absence of pathological findings indicates acute tolerability at 48 h after i.v. injection of 10 mg/kg Clarstatin in mice.

## 4. Discussion

We have previously [27] applied the cycloscan method to the shared epitope (SE) sequence -Gly-Gln-Lys-Cys-Gly-Ala-NH_2_ bridging the amide nitrogens of the two Gly residues with a urea-bridged, aliphatic bridge of various sizes and found a highly potent cyclic peptide with a ring size of 24 atoms. Moreover, we characterized a small SE-mimetic c(HS4-4), containing the SE primary sequence motif QKRAA, which was synthesized using a backbone cyclization method. The SE-peptidomimetic c(HS4-4) interacted strongly with the SE receptor calreticulin (CRT) [11]. The docking of c(HS4-4) to the CS-CRT:HLA-DRB1 complex revealed the importance of Gln70. We speculated, therefore, that replacing Gln in the SE-mimetic with Asp will lead to an effective inhibitor of HLA-CRT interaction. Indeed, bridging the amide nitrogens of the two Gly residues in the sequence -Gly-Asp-Lys-Cys-Gly-Ala-NH_2_ with a urea-bridged aliphatic ring of 11 atoms yielded a backbone cyclic peptide with a ring size of 24 atoms that potently abolished arthritis in mice [12]. With this background, in the present study, we designed, synthesized, and characterized another novel shared epitope (SE), thiourea-bridged backbone cyclic peptide, named Clarstatin, that reduced calcium levels in Jurkat lymphocyte cultures, was well tolerated in cell cultures upon acute toxicity testing, and ameliorated uveitis in vivo in an EAU mice model. To our knowledge, this is the first description of the SPPS of a backbone cyclic peptide with a thiourea bridge. We used the same strategy described by Touati-Jallabe et al. [22] for the SPPS, namely, we incorporated two building units having the Mtt and Alloc orthogonal protection on the N-alkyl amino groups. We found out that the on-resin generation of the isothiocyanate, after the removal of the Alloc protecting group, was best performed by the procedure described by Boas et al. [28] rather than the procedure described by Touati-Jallabe et al. [22]. Apparently, CS2 + HBTU gave better yields than di-2-pyridylthionocarbonate (DPT) for the generation of the isothiocyanate. Using these procedures, we obtained, after purification with prep. HPLC, a highly pure, biologically active peptidomimetic named Clarstatin.

Beyond offering new insights into the drug development of novel peptide biologics for uveitis, the present findings illustrate the therapeutic target ability of the HLA-DR ‘shared epitope’ sequence–calreticulin signaling pathway by a specific, rationally designed backbone cyclic peptide. Clarstatin showed potent activity both in vitro and in vivo. This finding could facilitate future optimization efforts of Clarstatin. From the medicinal chemistry perspective, it is also worth noting that the thiourea bridge and the introduction of two glycines did not appear to play a functional role in the compounds’ biological effects.

The main significance of the findings reported here relates to the fact that they propose Clarstatin as a novel biologic lead compound for the treatment of uveitis. To date, novel treatment modalities in uveitis such as monoclonal antibodies have targeted cytokines, their receptors, or other players in the immune-activated final common pathway [29]. Due to their involvement in the final steps of uveitis pathogenesis, the current treatment with biologics is less effective than steroids but safer [30]. The advantage of the Clarstatin approach over current or emerging drugs is that it addresses an unmet need by offering a potent intervention strategy that specifically targets the early important event in the lymphocyte pathologic autoimmune inflammatory cascade by targeting and inhibiting the interaction between the HLA-DR ‘shared epitope’ sequence and calreticulin. Calreticulin, in addition to being localized in the endoplasmic reticulum, is also expressed in other subcellular compartments such as the nucleus, the nuclear envelope, the cytosol, and the cell surface of the cells. Therefore, Clarstatin’s ability to reduce cytosol calcium levels can be attributed to its ability to antagonize calreticulin activity in one of these cellular compartments. A decreased calreticulin level will decrease the lymphocyte’s cytosol calcium levels as well as Ca^2+^ storage capacity and Ca^2+^ sequestering ability, thus causing increased sensitivity of cells toward the intracellular Ca^2+^ level and signaling [31]. This hypothesis is supported by findings indicating that treatment of cells with antisense nucleotides decreases calreticulin expression and lowers the Ca^2+^ response [32]. A decrease in cytosolic calcium in leukocytes may affect the cellular compartment distribution of calreticulin and may have implications for cytokine production and innate inflammatory purposes [33]. The present results clearly and indirectly indicate that the synthetic Clarstatin is active in vitro, most probably by functionally antagonizing the calcium-buffering ability of cellular calreticulin.

Experimental autoimmune uveoretinitis (EAU) is thought to be a representative model for the study of therapeutic approaches to human posterior uveitis [34]. EAU can be induced in animals through systemic immunization with retinal proteins, initiating an immune response that results in tissue damage. This process is orchestrated by CD4(+) T cells specific to autoantigens, whose activation triggers the infiltration of various other leukocytes into the retina. Throughout EAU, immune cells penetrate the eye’s parenchyma, causing pathological processes on both the retina and choroid. In the present study, it was found that treatment with Clarstatin reduces the severity of EAU, most probably reflecting reduced inflammatory cell recruitment and infiltration into the eye. Therefore, it is reasonable to propose that Clarstatin inhibited the process of inflammatory cell recruitment to the eye that involves their activation by elevation of the intracellular calcium [35], as evident from the present experiments with the Jurkat cells. The possibility that Clarstatin also inhibited adhesion, and/or migration, and eye infiltration, preventing focal retinal tissue damage, is under investigation in our laboratories.

## 5. Conclusions

This study suggests that Clarstatin could be a useful thiourea-bridged backbone cyclic SE peptidomimetic lead compound and tool for studying the mechanisms governing uveitis. Furthermore, the results of this study provide a rationale and early preclinical information that could pave the way for the development of specific, potent, safe, and inexpensive drugs for uveitis.

## 6. Patents

Gilon C, Hoffman A, Lazarovici P, Radgonde A. Calreticulin peptidomimetic inhibitors and prodrugs. U.S. provisional patent application No. 63/477,201 filed on 26 December 2022 and refiled 26 December 2023. Assigned to YISSUM research and development Co. of the Hebrew University of Jerusalem LTD., and HADASIT Medical Research services and development LTD.

## Data Availability

The data presented in this study are available in this article.
